# Are residents of downtown Toronto influenced by their urban neighbourhoods? Using concept mapping to examine neighbourhood characteristics and their perceived impact on self-rated mental well-being

**DOI:** 10.1186/1476-072X-11-31

**Published:** 2012-08-03

**Authors:** Amanda J Sheppard, Christina Salmon, Priya Balasubramaniam, Janet Parsons, Gita Singh, Amina Jabbar, Qamar Zaidi, Allison Scott, Rosane Nisenbaum, Jim Dunn, Jason Ramsay, Nasim Haque, Patricia O’Campo

**Affiliations:** 1 Centre for Research on Inner City Health, Keenan Research Centre, Li Ka Shing Knowledge Institute of St. Michael’s Hospital, 30 Bond Street, Toronto, Ontario, M5B 1W8, Canada; 2 AboutKidsHealth, The Hospital for Sick Children, 555 University Avenue, Toronto, Ontario, M5G 1X8, Canada; 3 Neurorehabilitation Program, Toronto Rehabilitation Institute, Toronto, Canada; 4 FutureHealth Inc., Toronto, Canada; 5 Applied Health Research Centre, Keenan Research Centre, Li Ka Shing Knowledge Institute of St. Michael’s Hospital, Toronto, Canada; 6 Department of Physical Therapy, University of Toronto, Toronto, Canada; 7 Department of Obstetrics & Gynecology, Markham-Stouffville Hospital, Toronto, Canada; 8 Dalla Lana School of Public Health, University of Toronto, Toronto, Canada; 9 Department of Social Sciences, Health Studies, University of Toronto Scarborough, Toronto, Canada

**Keywords:** Mental well-being, Neighbourhoods, Concept mapping, Qualitative methods

## Abstract

**Background:**

There is ample evidence that residential neighbourhoods can influence mental well-being (MWB), with most studies relying on census or similar data to characterize communities. Few studies have actively investigated local residents’ perceptions.

**Methods:**

Concept mapping was conducted with residents from five Toronto neighbourhoods representing low income and non-low income socio-economic groups. These residents participated in small groups and attended two sessions per neighbourhood. The first session (brainstorming) generated neighbourhood characteristics that residents felt influenced their MWB. A few weeks later, participants returned to sort these neighbourhood characteristics and rate their relative importance in affecting residents’ ‘good’ and ‘poor’ MWB. The data from the sorting and rating groups were analyzed to generate conceptual maps of neighbourhood characteristics that influence MWB.

**Results:**

While agreement existed on factors influencing poor MWB (regardless of neighbourhood, income, gender and age), perceptions related to factors affecting good MWB were more varied. For example, women were more likely to rank physical beauty of their neighbourhood and range of services available as more important to good MWB, while men were more likely to cite free access to computers/internet and neighbourhood reputation as important. Low-income residents emphasized aesthetic attributes and public transportation as important to good MWB, while non-low-income residents rated crime, negative neighbourhood environment and social concerns as more important contributors to good MWB.

**Conclusion:**

These findings contribute to the emerging literature on neighbourhoods and MWB, and inform urban planning in a Canadian context.

## Background

In 2003 and 2004, Canadian expenditures on mental health and addictions cost roughly $6.6 billion; while this amount is lower than in most industrialized countries, the economic as well as the personal and social costs are considerable [1]. Given the high costs associated with mental illness and the complexity of the problem, identifying pathways to promoting mental well-being represents a valuable contribution.

There is evidence that physical and social features of urban environments are associated with mental health. Aspects of light, indoor air quality, noise, crowding, housing, neighbourhood quality, and other features, all directly correlate to mental health [2]. Aspects of mental health (such as personal control and social support) have been found to be associated with the built environment (including exposure to natural elements and views of nature) [3]. In Evans’ review of these literatures, he argues for developing studies that go beyond observing associations, to those that attempt to understand the pathways of influence between these environmental factors and mental health [2]. Furthermore, these pathways may work differently among populations with different attributes. Groups with lower socioeconomic position are more likely to be depressed compared to those with higher socioeconomic position [4]. Accordingly, it would be essential to examine this and related characteristics.

Neighourhood-level characteristics have been studied specifically in an attempt to understand contextual pressures on mental health. Context-specific pathways, shaped by for instance, peoples’ lived experiences, age, and socioeconomic position, have been demonstrated to influence health [5,6]. A systematic review found a significant association between mental health and at least some aspect of neighbourhood influence in 93% of the papers that met their inclusion criteria [7]. Wen and colleagues employed both objective and subjective measures of neighbourhood environment to study their influence on individual health [8]. In order to explore the context-specific pathways which lead to poor health outcomes, new methodologies (ones which employ more sophisticated measurements and conceptualizations of neighbourhoods and public health outcomes) are required [9]. Traditional quantitative methods do not have the flexibility to capture the complexity of relationships between context-specific variables and person-centred variables. Alternative approaches are more likely to enable the development of a conceptual framework which will in turn yield testable hypotheses about specific relationships between person and place [10-13].

More recent studies have employed a structured, focused methodology termed “concept mapping” (CM) to investigate participant perceptions about a specific topic (e.g., factors in their neighbourhood that they feel impact their mental well-being (MWB)) to generate a meaningful framework which groups perceptions into cohesive concepts [14-18]. These latter two analyses engaged participants sampled from downtown and inner-suburban neighbourhoods to identify neighbourhood characteristics that influence MWB in a good or bad way [14,17]. O’Campo and colleagues’ (2009) findings revealed a few differences between the perspectives offered by participants from the downtown- and inner-city suburbs. For instance, those from the inner suburbs ranked a data cluster representing ‘necessary human and social services’ as more important to their good MWB compared to the downtown participants [17]. Burke et al. (2009) examined gender differences in perceptions of characteristics influencing MWB, but significant differences between genders were only identified after stratifying for socioeconomic status.

Mental health conditions are on the rise, paralleling an increasing proportion of the Canadian population living in urban centres. The most recent national census revealed that over 80% of Canadians live in urban centres, and the provinces of Ontario and British Columbia have about 85% of their population living in cities [19], strengthening the importance of investigating these city dwellers’ perspectives on neighbourhood characteristics that affect mental health.

The goal of this study was to uncover participants’ perceptions regarding the relationships between neighbourhood structural and social characteristics and mental well-being. We examined residents’ perceptions (varying by age, gender, individual income level, and neighbourhood socio-economic status) of which neighbourhood characteristics influenced their feelings of mental wellbeing. This study had two objectives: (1) To generate a list of neighbourhood characteristics that a sample of downtown Toronto residents feel influence their MWB in positive and negative ways and (2) explore differences in the rank importance of these characteristics on the basis of demographic and depression status differences between resident groups.

## Methods

### Study design

Concept mapping is *“a structured process, focused on a topic or construct of interest, entailing input from one or more participants, that produces an interpretable pictorial view (concept map) of their ideas and concepts and how these are interrelated*” [20]. Qualitative and quantitative methods are used in concept mapping by creating a visual display of how the participants and the group as a whole conceptualize a particular topic. Data gathering activities are completed by each participant (brainstorming, sorting and rating, mapping) in order to fully represent each individual’s viewpoint while also incorporating group consensus.

### Study population

Participants were recruited (1) by contacting community organizations in the neighbourhoods who posted flyers and encouraged clients from their organizations to attend, and (2) by posting flyers widely in public spaces such as community centres, grocery stores and public transportation outlets. Flyers invited participants to attend discussion groups about neighbourhoods and health and encouraged interested parties to contact the investigators by phone. Inclusion criteria were: residency within the specific neighbourhood of 6 months or longer; 18 years of age or older; proficiency in English (written and oral – this was required as concept mapping requires completion and manipulation of written material); availability to participate in a group session and preferably able to attend more than one of the sessions.

### Data collection and analysis

Data collection was comprised of two group-based activities: brainstorming and a separate session in which the items generated during brainstorming were sorted, rated and mapped. While descriptions of the concept mapping method and the specific data collection procedures are detailed elsewhere [16], we describe each step briefly here. The group data generation events were held at centrally located agencies (all accessible by public transportation) within each of the five neighbourhoods. Chosen neighbourhoods (four low income, and two non-low income) were those selected towards a larger study, the Toronto Intensive Research on Neighbourhoods & Health Initiative (Toronto-IRONhI). Before each brainstorming session, a short survey was administered to participants, collecting information on age, gender, income, and length of residence in their neighbourhoods. In addition, participants answered a question about whether they felt safe in their neighbourhood. Furthermore, participants completed a short screening instrument for depressive symptoms (CES-D) [21]. This was not intended to target participants who do or did have depressive symptoms; conversely it was done to measure whether participants who had more depressive symptoms might report/rate items differently than those participants who did not score high on depressive symptoms.

#### Brainstorming

Each brainstorming session lasted approximately 1.5 hours. Brainstorming groups involved free-listing of items around a focus prompt. Participants were asked to list “characteristics of your neighbourhood that can affect MWB in either a good or bad way.” Definitions of the terms “neighbourhood” and “MWB” were provided (see Table 1), to ensure consistency within and across groups. While we were interested in mental health broadly, we used the term MWB to ensure the concept was interpreted to include holistic aspects of mental health, not simply clinical or negative views. Once brainstorming was completed for each neighbourhood group, the list of items was merged. This larger list was manually edited so that duplicates were eliminated and like items consolidated to yield a master list of items to be used in the subsequent study activities [22].

**Table 1:** Working definitions and focal questions used in the study

	
Neighbourhood	A physically bounded area characterized by some degree of homogeneity and sometimes social cohesion.
Mental well-being (MWB)	MWB refers to the psychological well-being of a person. This may include positive mental states such as being satisfied with life, happiness, or being stress-free. MWB also includes poor psychological states such as being highly stressed, feeling anxious, being fearful, bored or unhappy. Finally, MWB can include mental illness like major depression or even substance abuse like alcoholism.
Rating question about Good/Positive MWB	Please rate on a scale of 1 to 5 how each of these neighbourhood characteristics is related to a person’s good or positive MWB.
Rating question about Poor/Negative MWB	Please rate on a scale of 1 to 5 how each of these neighbourhood characteristics is related to a person’s poor MWB and/or mental illness.

#### Sorting, rating and mapping

Subsequently, sorting, rating and mapping sessions were held in each study neighbourhood (3-4 hours duration each). For the sorting task, participants were handed cards that contained one of each of the items from the master list. Participants were asked to place the items into piles that “made sense to them” and label the piles accordingly. The participants worked independently grouping similar items together according to their own perspectives. Labels were given to the piles by each participant reflecting the theme linking the items in the pile.

Following sorting, each participant completed two rating activities. The items were listed on sheets along with two rating questions. Participants were instructed to rate items according to how strongly they perceived the items to be related to either: (1) good MWB or (2) poor MWB, using a 5 point scale (ranging from 1 = not related at all to 5 = strongly related).

Once sorting and rating were completed, the data were immediately entered into the Concept Systems software where multidimensional scaling and hierarchal cluster analysis were conducted on-site [22]. Information about the distance of each item to all other items was produced to illustrate “clusters” of items representing conceptual domains. The Concept Systems software performed these analyses quickly, allowing us to share the results with the participants during the latter half of the second group session. These joint analytic activities yielded a final group map which represented the group’s viewpoints.

After these group sessions, we had only group-specific representations of the concept map; meaning that each group only processed data from their own neighbourhood and did not have the benefit of seeing the cumulative data maps generated by the other groups due to time constraints. The researchers later processed the data further to arrive upon the final aggregated concept map. The content and labels of each cluster across the groups were compared. High levels of similarity were found. Stress values, a diagnostic statistic for concept map data calculated for group specific and overall map solutions [22], were compared and found to be virtually identical. Final maps included data from all study participants.

After completing the group sessions, further analyses were conducted through pattern matching, a method to compare the average cluster ratings for a single variable by single or multiple groups, incorporating data from the demographic and CES-D surveys. We stratified the sample by potential confounders such as age, gender, and income to assess whether differences by these characteristics were observed. We also examined differences based on CES-D scores.

This study was approved by the Research Ethics Board at St. Michael’s Hospital, in Toronto, Canada.

## Results

### Participant characteristics

Forty-two participants took part in the brain storming sessions and subsequently, 35 (83%) remained to be involved in the sorting, rating and mapping sessions. Among these 35 participants from five downtown Toronto neighbourhoods, 80% (n = 28) were low income with an annual household income of $20,000 or below, and 20% (n = 7) were non-low income with an annual household income of $21,000 and above. There were 9 men and 26 women in our groups. Five people were under age 30 and the remaining participants were age 30 or older. Age 30 was selected to ensure any youth were captured in the lower age category, as they may respond differently than ‘adults’. Approximately six hours were spent with each individual talking about and undertaking activities regarding neighbourhoods and MWB across all the group sessions in each neighbourhood.

### Neighbourhood characteristics

Table 2 presents our consolidated list of neighbourhood items from the five neighbourhoods that were obtained through the brainstorming process. The 400 non-unique items generated across the brainstorming activities were combined into one consolidated list of 70 items.

**Table 2:** Brainstorming statement sorted into their final cluster solutions

Item Name (item number*)	**Good MWB**	**Poor MWB**
**Cluster 1: Crime in neighbourhood**		
crime (40)	moderate	High
poverty (34)	low	High
visibility of drug trafficking or drug use (43)	low	High
police harassment (12)	low	High
vandalism (1)	low	High
gangs (4)	low	High
**Cluster 2: Social concerns**		
safety (27)	High	moderate
neighbourhood lighting (10)	High	moderate
police involvement with community (46)	High	moderate
gentrification (53)	moderate	low
isolation (37)	moderate	High
class discrimination (38)	low	High
fights between neighbours (29)	low	High
**Cluster 3: Negative neighbourhood environment**		
a visible marginalized population (e.g. homeless) (63)	moderate	moderate
overcrowded housing (51)	moderate	High
litter (2)	low	High
noise (30)	low	High
heavy traffic (21)	low	moderate
bad smells (58)	low	moderate
cigarette butts (33)	low	moderate
**Cluster 4: Creates positive community environment**		
sense of community (42)	High	moderate
residents being involved in community change (16)	High	low
friendliness (35)	High	moderate
neighbours interacting (25)	High	moderate
newcomer friendly (52)	High	moderate
community cohesion in a crisis (54)	High	moderate
people who know you (31)	High	low
multicultural and multilanguage neighbourhood (36)	High	low
diversity (e.g. age, income) (66)	moderate	low
public celebrations (6)	moderate	low
**Cluster 5: Community friendly environment**		
pedestrian friendly neighbourhood (70)	High	moderate
neighbourhood watch (44)	High	low
neighbourhood reputation (32)	moderate	moderate
pet friendly neighbourhood (45)	moderate	low
neighbourhood history (56)	moderate	low
**Cluster 6: Essential services in the neighbourhood**		
community centre with adequate services and resources (69)	High	moderate
employment opportunities (50)	High	moderate
quality of schools (18)	High	moderate
job placement and training services (3)	High	moderate
accessible and accountable politicians (65)	High	moderate
services and resources that are age, family, gender and culturally appropriate (48)	High	moderate
a wide variety of educational opportunities (e.g. ESL, adult education) (13)	High	low
businesses that provide mentorship and apprenticeship opportunities (62)	High	low
volunteer opportunities (9)	High	low
outreach and mobility of community services and resources (64)	High	moderate
a range of services for low income populations (e.g. food banks, community kitchens) (68)	High	moderate
**Cluster 7: Beneficial community services**		
libraries (60)	High	low
public places for a variety of physical recreational activities and sports (e.g. ice hockey, pool, soccer fields) (22)	High	moderate
accessible and affordable child care (24)	High	low
access to places of worship (mosque, temples, churches, synagogues) (47)	High	Low
a range of traditional and non-traditional health services (20)	High	Moderate
services to support homeless (23)	High	Moderate
free access to computers and the internet (57)	High	Low
**Cluster 8: Neighbourhood amenities**		
access to banking services (14)	High	Low
grocery stores with a wide selection of food choices (17)	High	Low
laundry facilities (39)	High	Low
assortment of restaurants (11)	High	Low
coffee shops (5)	High	Low
bars (55)	low	Low
beer and liquor stores (59)	low	Low
**Cluster 9: Causes that make a neighbourhood aesthetically pleasing**		
clean air (7)	High	Moderate
parks (15)	High	Moderate
amount of natural landscape (e.g. tree lined streets) (67)	High	Moderate
well maintained streets and sidewalks (28)	High	Moderate
well maintained businesses and housing structures (61)	High	Low
physical beauty (e.g. fountains) (19)	High	Low
mixed-use neighbourhood (commercial, residential and/or recreational use) (41)	moderate	Low
**Cluster 10: Transportation**		
access to public transportation (8)	High	Moderate
short commute times (49)	High	Moderate
adequate parking (26)	moderate	Low

**Figure 1:**
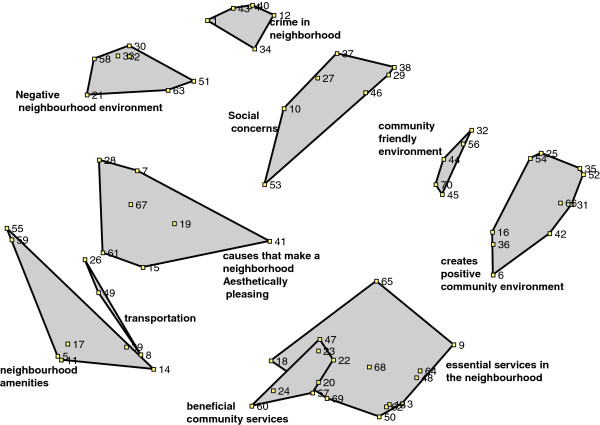
Cluster Map for the Relationship Between Good and Poor MWB, Ten Cluster Solution (n = 35).

The sorting activity, multidimensional scaling and the hierarchical cluster analyses yielded point maps and cluster maps. Of the 5 groups who participated in sorting and rating, only three neighbourhoods had sufficient time to review maps. The group members participated in the processing of the map data such that a consensus map was produced. In each of the groups where maps were reviewed, consensus maps contained on average 9-10 clusters. The final map, which contained data from all participants, was obtained by combining and consolidating across the group consensus maps. The final ten cluster concept map is presented in Figure 1. The items comprising the clusters are denoted by numbers in the figure that correspond to the item numbers in Table 2. Items that are closer together on the map are more closely related to one another as determined by the multidimensional scaling analyses. For example, item 29 “fights between neighbours” at the top of the Social Concerns cluster is closely related to item 38 “class discrimination” but is not as closely related to item 53 “gentrification” which is further down in that same cluster (Figure 1). The stress value for the map generated with all the data was 0.22, within the acceptable range reported in the concept mapping literature of most maps generated [22].

We categorized the items’ ratings for good MWB and poor MWB into high (3.8 and higher), moderate (2.9 to 3.7) and low (2.8 or lower) [18]. These ratings are presented in Table 2. Some examples of items that were rated as highly important for good MWB were “safety”, “access to public transportation”, “quality of schools”, “parks” and “friendliness”. Some examples of items that were rated as highly important for poor MWB were “crime”, “isolation”, “poverty”, “class discrimination”, “litter” and “police harassment”. Overall, almost four times as many items were rated as highly important to good MWB as to poor MWB, suggesting more agreement as to what contributes good MWB compared to poor MWB.

**Figure 2:**
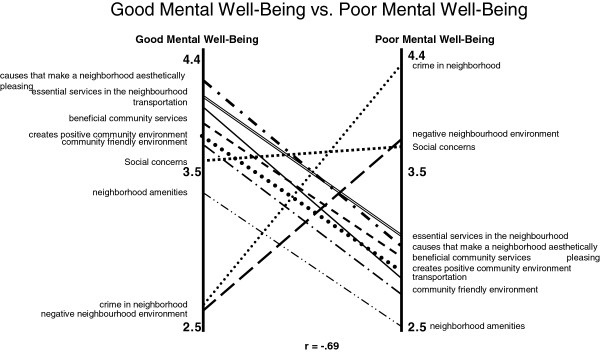
Pattern match of clusters in relation to Good vs. Poor MWB (n = 35).

**Figure 3:**
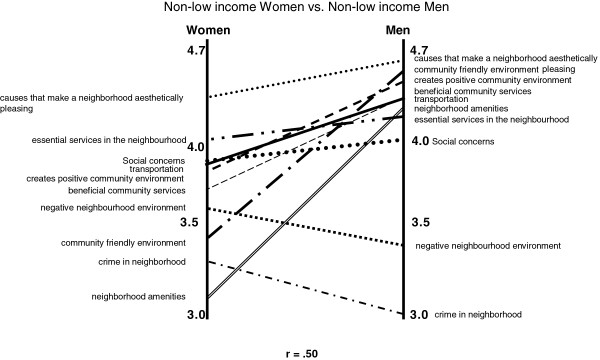
Pattern Match – Non Low Income Men vs. Non Low Income Women for Good MWB (n = 7).

**Figure 4:**
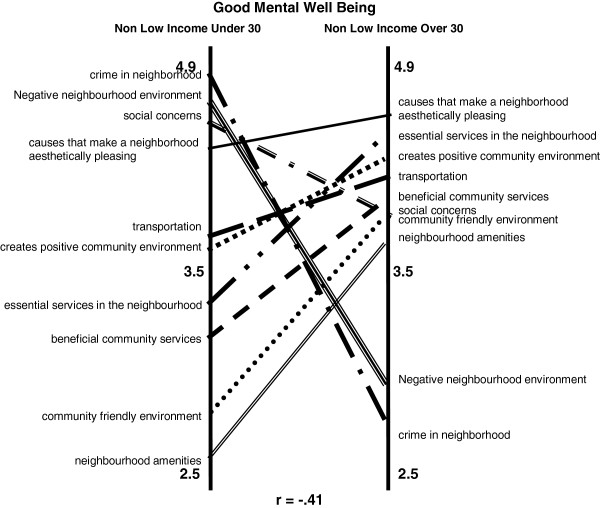
Good MWB in those Under Age 30 vs. Over Age 30 (n = 7).

Another way we examined the rating data was via pattern matching. Here, average ratings for each cluster are graphed. In Figures 2, 3 and 4, ratings for both good and poor MWB are contrasted in pattern matching by various demographic characteristics and correlations are noted within the figures. In Figure 2 the pattern match for good versus poor MWB is presented. This examination demonstrates for example, “causes that make a neighbourhood aesthetically pleasing” was strongly related to good MWB, while only moderately related to poor MWB. Conversely “crime in neighbourhood” was most strongly associated with poor MWB.

### Influence of income, gender and age

Differences in the average cluster rating for good MWB between those with low income versus non-low income were minimal (correlation 0.88, suggesting little difference between the groups) (data not shown). However, when we stratified within the non-low income ratings for good MWB by gender, differences were observed (correlation 0.50) (Figure 3). Non-low income men assigned a higher rating for clusters such as “community friendly environment” and “neighbourhood amenities” compared to women. Non-low income women gave higher ratings to clusters such as “essential services in the neighbourhood” and “social concerns” compared to men. As we stratified further we acknowledged that we were comparing small numbers. No such differences were seen for the ratings of poor MWB.

No substantial rating differences were seen when we stratified by age (<30 versus ≥30) (data not shown). However, when we examined age categories within the non-low income strata, we did see differences with a correlation of 0.41 (Figure 4). “Crime in a neighbourhood” and “negative neighbourhood environment”, were rated highest for individuals less than 30 years old in the non-low income group compared to being ranked lowest for individuals over age 30 in the same income group. “Causes that make a neighbourhood aesthetically pleasing” was rated first for individuals over age 30.

### Influence of depression status

Based on participants’ CES-D scores, we examined the extent to which depression (a score of 16 or higher on the CES-D) affected their ratings of neighbourhood items for both good MWB and poor MWB. In our sample, 19 participants did score 16 or higher. Correlations for those who were versus those who were not depressed were 0.95, suggesting no major differences in rating (data not shown).

## Discussion

Building on existing literatures that examined residents’ perceptions of neighbourhood characteristics that impact their MWB using concept mapping [14,17], our work is the first to examine these relationships while considering the influences of age and depression status. We identified a clear set of neighbourhood attributes that our participants perceived to be influencing MWB. In the 5 participating neighbourhoods, most respondents emphasized that aesthetic attributes, social and physical environment, accessibility, and availability of a range of services within the neighbourhood were all seen as important to their good MWB. Conversely crime and negative environmental attributes were seen as contributors to poor MWB. Attributes that *cause a neighbourhood to be aesthetically pleasing,* were of interest, because it was both the most subjective and least well defined concept to arise from the mapping sessions.

Heterogeneity within neighbourhoods was emphasized by residents, not uniformity. Those features contributing to poor MWB indicated that although residents wanted a diverse community, there was a point at which certain features could become problematic (e.g. while close proximity to neighbours was often seen as positive, it could give way to overcrowded housing and excessive levels of traffic and noise). While having a visible marginalized population (e.g. homeless) was seen as a negative feature, residents also emphasized the need for services to support the homeless and marginally housed within their communities.

We found considerable agreement among participants regarding features that were perceived to contribute to poor MWB. *Crime, negative neighbourhood environment (including overcrowded housing, noise, litter, heavy traffic) and social concerns (e.g. safety, isolation, class discrimination, gentrification)* were consistently cited as the three most important clusters influencing poor MWB, consistent with data from the United Kingdom [23]. This held true regardless of gender, household income, and age. Using successive waves of British crime data, Markowitz and colleagues (2006) suggest that there is a feedback loop acting in progression between several of these negative neighbourhood characteristics. Their model proposes that decreased neighbourhood cohesion leads to increased crime, promoting increased levels of fear which in turn decreases neighbourhood cohesion [24]. It is possible that similar feedback loops may be operating in the Toronto context.

There was less agreement among participants as to which features are most important to good MWB. As did Burke et al., we identified differences in how clusters were rated based on income status [14]. Low-income participants were more likely to indicate that aesthetic attributes (e.g. physical beauty, parks), availability of beneficial community services (e.g. public recreational facilities, libraries, health services, access to places of worship), and accessible transportation were influential to good MWB. Nielsen and Hansen suggest that *access* to a green area from an individual’s residence is associated with less stress, although the *frequency* of visits to a green area, was not associated with any health benefits [25]. Perhaps ‘just knowing’ the green area and/or other community services are available is important for good MWB among low-income participants. The importance of ‘knowing’ neighbours to visit, with whom to borrow or exchange favours has been shown to protect MWB in areas of deprivation in a multilevel population analysis in Wales [26]. This aspect of trust in other members of the community to explain health inequalities is supported by other Canadian data [17,27]. By contrast those experiencing higher family incomes and are younger than the age of 30 years old cited crime, negative neighbourhood environment, and social concerns as having the greatest influence on good MWB, whereas these were ranked very low among the older sample (Figure 4). Figure 3 demonstrates the contrast between the income groups; the contrasts are much less pronounced between men and women and between depressed/non-depressed participants.

The reasons for the income-related differences remain unclear. Perhaps higher-income individuals are more concerned with private property (i.e. more likely to possess it and have more to lose as victims of property crime). These concerns may in turn manifest themselves in anxiety and fear. A review of the literature of neighbourhood characteristics and maternal and child health reported that over one-third of the articles (n = 31) included a measure of residents’ perceptions regarding issues of social resources within their neighbourhoods [28].

Among the non-low-income participants, age appeared to have an effect on cluster ratings related to good MWB. The pattern matches in Figure 4 show that those participants under age 30 prioritized *crime, negative neighbourhood environment and social concerns* as highly important to good MWB – as opposed to their over-age-30 counterparts, who emphasized *neighbourhood aesthetics, the presence of essential services, and a positive community environment*. The reasons for such age-related differences remain unclear. These findings may reflect individual experiences rather than neighbourhood-specific ones. It will require further study to determine if younger respondents are more influenced than older residents by experiences of their peers (rather than their experiences of neighbourhood per se). Interestingly, for low-income groups, the age effect disappeared, showing considerable agreement on cluster ranking for good MWB.

While our findings varied somewhat based on participants’ demographic characteristics, they sketched complex linkages between neighbourhood features and residents’ MWB. Despite the small sample size, these data demonstrate that the contributors to good MWB are not simply a corollary of factors contributing to poor MWB. As such, this study represents a unique contribution to the existing literature on neighbourhoods and health.

A number of interesting questions were raised in the concept mapping sessions. We had assumed at the beginning of our study that built environment (as reflected by the exterior of buildings) was what constituted one of the primary physical features of a neighbourhood. This was challenged by participants during the brainstorming sessions. Those living in high-rises classified spaces within buildings (and between individual home units) as important features of neighbourhood. The importance of ‘internal spaces’ to positive MWB is further corroborated in the literature [2].

It is unlikely that employing a single methodology would be sufficient to capture the complex linkages between neighbourhood features and the MWB of residents. In order to fully understand the pathways by which neighbourhood features influence MWB, multiple methods are required [9]. In addition, perceptions of factors that are related to MWB may be different in Canadian neighbourhoods as compared to similarly sized American neighbourhoods. In a related study, our research team evaluated a composite systematic social observation (SSO) tool in these same neighbourhoods. This instrument is premised on the assumption that physical and social disorder negatively impact residents’ health that was derived from blockface observation tools pioneered primarily in the U.S.A. This SSO investigation contributed to our understanding of ‘neighbourhood disorder’ and led us to question whether this is the appropriate construct for the Toronto context [29]. This failure could be due to the nature of the tool, or differences between US and Canadian cities, or both. Therefore, the theoretical framework developed from concept mapping enhances our understanding of these relationships by revealing linkages between neighbourhood features (parks, tree lined streets, well maintained streets and sidewalks, mixed use of neighbourhood structures) and residents’ perceptions regarding their contribution to MWB. Our study questions the homogeneity with which area level factors have been judged to impinge on MWB. It suggests that it is important to acknowledge the differential impact of neighbourhood factors by demographic variables such as gender and income, newcomer status, and age.

This study has a number of limitations. We were only able to conduct interactive mapping sessions in 3 of the 5 neighbourhoods. The relatively low sample size resulted from difficulties in recruitment and retention of participants for subsequent group sessions. The sample was characterized by a skewed distribution in terms of age and gender – and by a preponderance of low-income participants and those not in the work force. Relatively few home owners and parents of young children were represented. Also, the high proportion of participants with relatively high depression ratings on the CES-D may also suggest a selection bias. The cross-sectional nature of this study failed to adequately capture residents’ experiences with neighbourhoods previously inhabited and how this might have shaped their perceptions of their current neighbourhoods.

The study has implications for other areas of research involving neighbourhoods, such as those using SSO methods. These data suggest that we need to redefine traditional constructs of neighbourhoods, for example looking at spaces within multi-residence buildings and residents’ perceptions of neighbourhood boundaries. Our data indicated that the relationship of neighbourhoods to MWB is one characterized by complexity – for example, residents spoke eloquently to both positive and negative aspects of gentrification, a process they saw as both potentially beneficial and harmful; in the end it was placed in the cluster of social concerns, a cluster more likely to contribute to poor MWB than to good.

## Conclusions

This study explored the complexities of neighbourhoods and health, and provided a more extensive framework for construing the relationship between neighbourhood characteristics and MWB in an urban Canadian setting. The research conceptual framework developed from this study will inform further investigation into urban planning and health policy in the Canadian context.

## Abbreviations

CM, Concept mapping; MWB, Mental well-being; SSO, Systematic social observation.

## Competing interests

The authors declare that they have no completing interests.

## Authors’ contributions

AJS participating in the design of the study, data collection and analysis, and drafted the manuscript. CS, PB, JP, GS, AJ, QZ, AS, RN, JR and NH all participated in the design of the study, data collection and analysis, and assisted with the manuscript preparation. JD and PO conceived the study, directed its design and coordination, contributed to the analyses and assisted with manuscript preparation. All authors read and approved the final manuscript.
